# Job Crafting and Burnout as Predictors of Food Safety Behaviors in the Foodservice Industry

**DOI:** 10.3390/foods11172671

**Published:** 2022-09-02

**Authors:** Leticia Guimarães Perdomo Nascimento, Ageo Mario Candido da Silva, Elke Stedefeldt, Diogo Thimoteo da Cunha

**Affiliations:** 1 Department of Food and Nutrition, School of Nutrition, Federal University of Mato Grosso (UFMT), Cuiabá 78060-900, Brazil; 2 Collective Health Institute, Federal University of Mato Grosso (UFMT), Cuiabá 78060-900, Brazil; 3 Department of Preventive Medicine, Universidade Federal de São Paulo (UNIFESP), São Paulo 04039-032, Brazil; 4 Multidisciplinary Food and Health Laboratory, School Applied Science, State University of Campinas (UNICAMP), Limeira 13484-350, Brazil

**Keywords:** food service, work engagement, food behaviors, training, restaurant, job demands

## Abstract

This study aimed to investigate whether job crafting, burnout, and work engagement predict food safety behaviors in the foodservice industry. It was a cross-sectional study conducted in Cuiabá (Brazil) among foodservice workers. Four instruments were used among foodservice workers for the examination: (a) job demands and resources, (b) job satisfaction, (c) burnout, and (d) work engagement. Food safety practices were measured using a validated risk-based checklist. Partial least squares structural equation modeling was used to test the hypothesis model. In this study, 22 restaurants and 302 foodservice workers were examined. It was found that the “job demands-resources” model was valid for foodservice workers, i.e., burnout was strongly predicted by job demands (β = 0.550; *p* < 0.001); job resources were a positive predictor of work engagement (β = 0.258; *p* < 0.001); and burnout was a negative predictor of work engagement (β = −0.411; *p* < 0.001). Food safety violations were predicted by job crafting (β = −0.125; *p* = 0.029) and burnout (β = 0.143; *p* = 0.016). The results indicate that mitigating burnout and increasing job crafting can be important supporting strategies to improve food safety behaviors.

## 1. Introduction

Globally, 420,000 people die each year from foodborne diseases (FBDs), 77,000 of them in the Americas alone (World Health Organization, 2015) [1]. In addition to their impact on health, FBDs also affect the economy. According to a World Bank report, low- and middle-income countries suffer a loss of 95 billion USD per year due to FBDs. The cost of treating FBDs is estimated at 15 billion USD annually [2]. It is widely recognized that it is necessary to promote safe food production to prevent FBDs, which is achieved through safe food-handling practices [3,4]. The most common factors reported in outbreaks with known causes are improper cooking, contaminated ingredients, contaminated equipment and utensils, cross-contamination, improper storage, and poor personal hygiene [5,6]. Based on the knowledge–attitude–practice approach, FBD prevention has traditionally focused on training employees in food handling [6,7]. The theory assumes that knowledge can promote positive attitudes and that attitudes, in turn, shape practices [7]. Although some authors advocate such a model [8,9], many have addressed its volatility [10,11]. Therefore, new strategies have been developed to understand safe food-handling practices. It is known that other factors also play an essential role [12], for example, organizational factors such as food safety culture [13,14,15], stress [16], burnout, job motivation, job satisfaction [17], and working conditions [18].

Research has shown that many variables relative to the work environment and employees have a significant impact on work performance, such as work engagement [19,20] and satisfaction [21], or on the other hand, tension, stress, or burnout [22], which negatively affect well-being [23] and performance [24]. Since work engagement is a fruitful state that benefits both the worker and the organization, and burnout is a counterproductive state with antagonistic effects on workers’ engagement and health, efforts have been made to examine factors that might influence such aspects [25]. Therefore, these conditions and variables can be related to foodservice worker’s behavior, including food safety behaviors, because appropriate food safety practices are among the many factors attributable to workers from this industry.

Several studies, including those in the restaurant industry, have shown that employee engagement contributes to their health [26,27,28] and performance [20,26,27]. In a study conducted in a fast-food chain in the U.S., researchers showed that employee engagement positively influenced their performance and contributed to good customer perception of the service provided [28]. To investigate how organizational and psychosocial variables influence worker behavior, the concept of job crafting has emerged in the current literature. Job crafting has been defined as the autonomous physical and cognitive changes that workers make to tasks or relationship boundaries in their work [29]. In practice, this refers to the actions individuals take to personalize and reshape their work to achieve a better person–role fit, leading to positive outcomes such as satisfaction, work engagement, and resilience.

Yet, it is not clear if and how job crafting might affect food safety practices. This is the first study to understand how job crafting may affect food safety practices in restaurants. It may also be promising to know how job demands and resources affect job crafting when considering all the aspects of such a complex environment. Applying organizational models, such as the Job Demands–Resources (JD-R) model, to predict food safety could prove fruitful in designing practices and training managers and employees in the foodservice industry. Thus, this study aimed to investigate whether job crafting, burnout, and work engagement predict food safety behaviors in the foodservice industry.

## 2. Model and Hypotheses

This study extends the JD-R model by adding job crafting as a predictor of food safety behavior. We propose seven hypotheses grounded on the current literature to study this model.

### 2.1. JD-R Model

The JD-R model, introduced in 2001, was extended to include self-reinforcing paths, such as job crafting [30,31]. This model assumes that all job characteristics can be classified into two categories, job demands and job resources, which have different effects. The first relates specifically to burnout, while the second refers to engagement [31].

Job demands are the physical, social, or organizational aspects of work that require sustained physical or mental effort and thus impose significant physiological and psychological effort. Examples of job demands include role conflict, workload, pressure at work, and unfavorable shift work. On the other hand, job resources are physical, psychological, social, or organizational aspects that help employees achieve goals, reduce job demands with physiological and psychological costs, or promote growth, learning, and personal development [32]. Examples include autonomy, justice, performance feedback, job security, decision-making participation, and supervisor and co-worker support [33].

According to the JD-R model, burnout develops in a scenario of imbalance between demands and resources at work, e.g., extreme demands leading to constant overload and exhaustion, combined with scarce resources to meet the demands [34]. The opposite is true, i.e., resources lead to a motivational process that results in greater engagement in work [35]. Burnout is a process of progressive suppression of energy and enthusiasm at work. It is characterized as a syndrome with three main dimensions: overwhelming exhaustion, feelings of cynicism, and sense of ineffectiveness [36]. Burnout begins with exhaustion in response to overload due to high demands without an equal monitoring of resources with a compensatory effort to maintain performance [37,38]. When this condition persists, the employees feel that their energy is depleted, causing them to withdraw from both colleagues and customers to preserve the resources that are still available. Due to the exhaustion and withdrawal behavior, the employee is often disapproved by colleagues, and the demands accumulate, making the development of effective work increasingly unlikely and gradually creating a sense of professional inefficiency, i.e., resource loss spiral [39,40].

A resource decline affects both burnout and work engagement [41]. Over time, studies have confirmed the importance of resources in increasing work engagement [42,43,44]. Thus, work engagement is reduced when the worker has access to fewer and fewer resources. In the global literature, burnout is negatively associated with engagement [45,46,47,48], i.e., as burnout increases, work engagement decreases. In a systematic review, an analysis of 12-month longitudinal studies found that burnout negatively predicted engagement at work [49].

There is a discussion about the difference between generic and specific demands of occupations or positions in the JD-R model [50]. Although it is a generic model, it is essential to test whether the proposed factors for evaluating demands and resources are consistent with job characteristics. Confirming a JD-R model in a specific population is one of the ways to assess how the different factors can be used in this context. Thus, we have the following assumptions:

**Hypothesis** **1** **(H1).** *Job demands positively predict burnout.*

**Hypothesis** **2** **(H2).** *Job resources positively predict work engagement.*

**Hypothesis** **3** **(H3).** *Burnout negatively predicts work engagement.*

Testing these hypotheses is necessary to check the JD-R model’s adherence to the foodservice context. In addition, no studies were found that used the JD-R model to explain food safety behavior.

### 2.2. Job Crafting

The concept of job crafting was developed based on and complements the classical job design theory [29]. According to Wrzesniewski and Dutton (2001) [29], job crafting refers to the upward proactive behavior of workers who seek to adapt their work to make it more meaningful, satisfying, and engaging or optimize their resources and demands [51,52]. Unlike job design, job crafting is not about specific agreements defined by the organization but about proactive change [53]. There are three job-crafting dimensions: task crafting, cognitive crafting, and relational crafting [29]. The first dimension refers to changes in the boundaries of the tasks that workers have developed in their workplace, which may refer to the number, scope, or nature of tasks. The second dimension, cognitive crafting, involves changes in the cognitive boundaries of the job that lead to a different perception of the job tasks and relationship. The last dimension involves changes in the relationships that occur at work, both qualitative and quantitative [54]. According to this understanding, job crafting helps workers reframe the purpose of their work, for example, adapting it to their passions and needs, resulting in more meaningful and satisfying work for them. In the JD-R context, job crafting improves job resources [29]. 

Research shows that job crafting generally benefits both the company and the employee. Those who engage in more job crafting experience better person–job fit [55], engagement [56,57], satisfaction [58], and even better health [59,60]. The relationship between job crafting and burnout is also the subject of research. Job crafting can act as either a moderator or a mediator between work and burnout by reducing the impact of the demands on workers’ health, thereby reducing the likelihood of burnout occurring [21,61].

Thus, it is plausible that job crafting can predict positive outcomes such as job resources and work engagement. In contrast, job crafting may be negatively correlated with burnout. With this in mind, we hypothesize:

**Hypothesis** **4a** **(H4a).** *Job crafting positively predicts job resources*.

**Hypothesis** **4b** **(H4b).** *Job crafting positively predicts work engagement*.

**Hypothesis** **4c** **(H4c).** *Job crafting negatively predicts burnout*.

### 2.3. Factors Affecting Food Safety Behavior

Many authors have been dedicated to studying the organizational aspects affecting food safety behavior [13,14,15]. This study understood food safety behavior as the frequency and risk associated with various food safety violations committed by foodservice workers, i.e., a practice-based definition. 

Job crafting may increase job performance [62]. In a survey of managers in 235 restaurants in South Korea, job crafting was found to be positively related to restaurant sales performance, in a win–win relationship, i.e., everyone wins, including the company itself, when individuals themselves make changes to the way they work to increase their satisfaction and work integration [63]. Through this proactive redesign of the workplace, i.e., job crafting, foodservice workers might more efficiently manage their tasks and develop a greater awareness of food safety. However, food safety behavior is a complex phenomenon. Food safety compliance is mainly seen as an additional requirement rather than a natural part of food handling, so it tends to be reactive rather than proactive [18]. In this case, burnout and work engagement may also play an essential role in food safety behavior. 

Burnout is known to have numerous effects on workers’ health [64], including cardiovascular disease, sleep disturbances, chronic fatigue, depression, anxiety [65,66], and an increase in workplace accidents [67]. At the organizational level, burnout leads to absenteeism, turnover intentions, and a decline in service quality and productivity [68,69]. High levels of burnout are prevalent among foodservice workers; however, burnout did not predict food safety behaviors in those studies [17,70,71]. Although some findings are controversial, there is general agreement that burnout, particularly exhaustion, is a negative predictor of job performance [72].

The positive counterpart of burnout, work engagement, is a positive and gratifying state related to work [36], characterized by vigor, dedication, and absorption [73]. “Vigor” refers to a high level of energy and mental resilience, “dedication” to a high level of engagement in work, and finally “absorption” to full concentration so that time passes quickly. In addition to contributing to employee health [74,75,76], several studies, including in the foodservice industry, have shown that employee engagement is positively related to employee performance [20,26,27]. Based on those assumptions, we hypothesize:

**Hypothesis** **5** **(H5).** *Job crafting negatively predicts food safety violations*.

**Hypothesis** **6** **(H6).** *Burnout positively predicts food safety violations*.

**Hypothesis** **7** **(H7).** *Work engagement negatively predicts food safety violations*.

**Figure 1:**
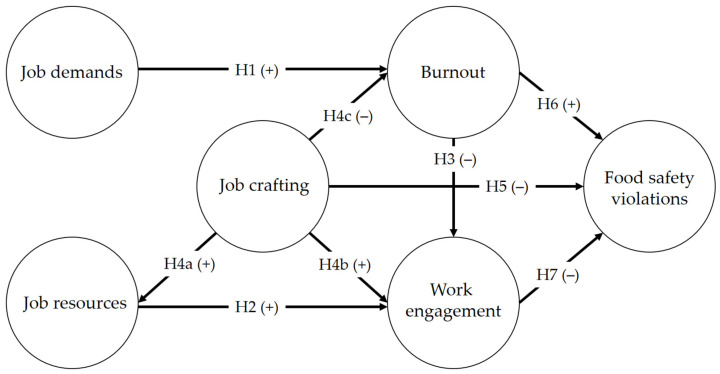
Hypothesis model.

Figure 1 depicts the hypothesis model.

## 3. Methods

### 3.1. Sample

This is a cross-sectional study conducted in the city of Cuiabá (state of Mato Grosso, Brazil) among foodservice workers working in small (up to 300 meals/day), medium (from 301 to 1000 meals/day), and large (more than 1001 meals/day) establishments.

Purposeful sampling is calculated for studies with structural equation modeling, using a predicted effect size of 0.3 and statistical power of 0.8, considering 5 latent variables and 26 observable variables [77]. A minimum sample of 261 subjects was required in this manner. By adding 15% due to possible dropouts, 302 foodservice workers were recruited in 22 restaurants. Only foodservice workers that were food handlers were invited, i.e., all persons who come into direct or indirect contact with food in the foodservice. The collection took place from February to March 2022 and was conducted by four academics and two researchers trained for this purpose. They all had experience in food safety. The Ethics Committee of Federal University of Mato Grosso approved the study (CAAE 53319421.3.0000.8124).

### 3.2. Measures

Four instruments were used among foodservice workers for the investigation, (a) job demands and resources, (b) job crafting, (c) burnout, and (d) work engagement, in addition to collecting the sociodemographic data of foodservice workers. The food safety behavior was evaluated through the direct observation of foodservice and food handling. Questions regarding foodservice documentation were answered by the manager or owner of the establishment.

a. Job demands and resources

Job demands and resources were assessed based on *Questionnaire sur les Ressources et Contraintes Professionnelles* (QRCP) [78] and Job Demands–Resources Questionnaire (JD-RQ). The questionnaire consisted of 28 questions, 14 related to job demands and 14 to job resources. The latent variable job demands consisted of three dimensions: pace and amount of work (4 indicators), physical effort (4 indicators), and role conflict (4 indicators). The latent variable job resources consisted of three dimensions: relationship with superior (4 indicators), justice (4 indicators), and relationship with colleagues (4 indicators). Indicators were answered using a 7-point Likert scale ranging from 1 (never) to 7 (always) [78].

b. Job crafting

To assess the latent variable job crafting, the Job Crafting Questionnaire (JCQ) [79], according to Slemp and Vella-Brodrick (2013) [80] was used. The JCQ consisted of 15 indicators distributed across three subscales: cognitive crafting (5 indicators), task crafting (5 indicators), and relational crafting (5 indicators). These indicators were answered on a 6-point Likert scale ranging from 1 (rarely) to 6 (often) (adapted from Devotto and Machado (2020) [79]).

c. Burnout

Burnout was assessed with the Maslach Burnout Inventory-General Survey (MBI-GS) [39]. MBI-GS consists of 16 indicators across three dimensions: professional efficacy (6 indicators), cynicism (5 indicators), and exhaustion (5 indicators). The version of MBI-GS with cross-cultural adaptation and validation for Portuguese was used [81]. The questions were answered on a 7-point Likert scale, ranging from 0 (never) to 6 (every day). Cutoff points based on tertiles were used to classify the burnout level for each dimension [82]. Burnout level was classified as low (score ≤ 1.62), medium (>1.62 score ≥ 1.87), and high (score > 1.87). MBI-GS is a copyrighted instrument by Mind Garden (<www.mindgarden.com> accessed on 16 July 2022). Appropriate licenses to use and reproduce were acquired.

d. Work engagement

Work engagement was assessed using the Utrecht Working Engagement Scale (UWES) [73], which was adapted and validated for the Brazilian population [83]. The scale contains nine indicators divided into three dimensions: vigor (3 indicators), dedication (3 indicators), and absorption (3 indicators). These indicators were answered on a 7-point Likert scale ranging from 0 (never) to 6 (always). According to the UWES manual [84], the average engagement at work and its subscales can be classified as very low, low, medium, high, and very high based on the cutoffs described in this manual.

e. Food safety violations and behavior

The researchers observed employee practices during a workday. Food safety violations were assessed using a validated risk-based checklist [85]. The checklist contained 50 indicators of food safety violations in nine sections: 1—water supply; 2—construction, facilities, equipment, furniture, and utensils; 3—sanitization of facilities, equipment, furniture, and utensils; 4—integrated control of disease vectors and urban pests; 5—food handlers; 6—raw materials, ingredients, and packaging; 7—food preparation; 8—storage and transport of prepared food; and 9—documents. The answers to the questions were adequate, not adequate (violation), and not applicable. Each item had a specific score based on the likelihood and consequence of the violation. The overall risk score ranged from 0.0 to 2498.6 with a negative magnitude, i.e., the higher the score, the higher the risk of FBDs. Based on the score, food services were classified into five groups: group 1 (0.0)—lowest risk; group 2 (0.1–13.2); group 3 (13.3–502.6); group 4 (502.7–1152.2) and; group 5 (above 1152.2)—highest risk [85]. To define food safety behavior, we included a latent variable with three sections (5, 6, and 7), directly related to employees’ practices, such as hand washing, cross-contamination prevention, cooking practices, and others. This was a group variable, i.e., if one foodservice worker committed a violation, the entire group received the risk score. This procedure has been used in previous studies [17,86], and limits the individualization of organizational outcomes.

### 3.3. Analysis

All variables were subjected to the Shapiro–Wilk normality test, and the variables met the assumptions of nonparametric distribution. After classifying the data distribution, a descriptive analysis was performed using the measure of central tendency (median) and dispersion (interquartile range) in addition to the distribution of frequencies. Partial least squares structural equation modeling (PLS-SEM) was used to test the hypothesis model. SEM allows models with a large number of independent and dependent variables to be elaborated. PLS-SEM was chosen because it is more appropriate for variables measured with a Likert scale and for non-normal data and allows complex analyses to be conducted without large samples [87,88]. While covariance-based structural equation modeling is more suitable for theory confirmation, PLS-SEM is better suited for predicting key target constructs [87], such as the hypotheses in this study. The model constructs (job demands, job resources, job crafting, burnout, work engagement, and food safety violations) were obtained by including the dimensions’ variables, i.e., the scores of the indicators were summed to create new variables of the dimensions. Before summation, the composite validity of each dimension and the factor loading of each indicator were checked using a confirmatory factor analysis.

The measurement model was assessed through factor loadings (>0.40), composite reliability (CR > 0.80), and the average variance extracted (AVE > 0.40). The discriminant validity of the model was assessed using the cross-loadings, which are considered appropriate when the indicators have higher factor loadings in their respective latent variable than the factor loadings presented along with the other latent variables [89]. Multicollinearity was assessed using the variance inflation factor (VIF) value (<5.0). The structural model was assessed through the variance explanation of the endogenous constructs, effect size (f^2^ > 0.15), and predictive relevance (*Q*^2^*_predict_*). The effect size (f^2^) was classified as small (f^2^ ≥ 0.02), medium (f^2^ ≥ 0.15), or large (f^2^ ≥ 0.35) [90]. A bias-corrected bootstrapping procedure with 5,000 samples was used to estimate the t-statistics and the *p*-values (significance: *p* < 0.05) of the estimated loadings. Student’s t-test was used to compare two independent groups. Data were analyzed using Stata v. 16.0 (Stata Corporation, College Station, TX, USA) and Smart PLS v 3.2.8 (Ringle, Wende, and Becker, 2015).

## 4. Results

### 4.1. General Results

In this study, 22 restaurants were investigated. Of these, 50% were small establishments with up to 300 meals per day and an average of 9.1 employees; 40.9% were medium-sized establishments (301 to 1000 meals) with an average of 19.5 employees; and 9.1% were large establishments with more than 1000 meals per day and an average of 38.5 employees. Regarding the risk of foodborne illness, 12.2% of restaurants were classified in group 3, 36.4% in group 4, and 45.5% in group 5, i.e., the groups at the highest risk of FBDs.

Table 1 shows the mean FBD risk score and the mean percentage of violations. 

The section with the highest percentage of violations was 5 (food handlers), with a mean violation of 77.26% (Table 1). This section included food handler hygiene, hygienic behavior (e.g., do not smoke and speak when unnecessary and do not sing, whistle, sneeze, spit, cough, eat, handle money, etc.), and employee health. The second section with the highest percentage of violations was Section 3 (sanitization of facilities, equipment, furniture, and utensils), with 55.44%.

Table 2 describes the socioeconomic and labor profile of the foodservice workers. The foodservice workers who participated in this study (*n* = 302) were evenly distributed between sex (51.32% men) and marital status (52.65% single) and were on average 33.66 years old. It was also found that the majority (60.26%) had completed at least high school and that 35.1% of the employees had not attended any food safety training. The average experience of these employees was less than ten years (9.7 years), and only an average of 3.5 of those years were spent in the current foodservice.

### 4.2. Measurement Model

As shown in Table 3, the reliability and validity of the constructs were tested using AVE, CR, and loading factor. The constructs job demands, job resources, burnout, and work engagement had AVE values greater than or equal to 0.50, indicating sufficient convergent validity. According to Fornell and Larcker (1981) [91], values of AVE less than 0.5 up to a limit of 0.4 are accepted as long as CR is greater than 0.60. In this study, the AVE value of the job crafting variable was 0.735, so it could remain in the model. All factor loadings were above 0.48, and most factor loadings, 67% (10), had values above 0.75. The CR values of all latent variables were between 0.7 and 0.9, which, according to Hair et al. (2021) [89], is considered satisfactory. All indicators had VIF values of less than 2.35, which is well below the recommended parameter (VIF < 5) and shows no problems with multicollinearity.

All dimensions were similar between sexes (*p* > 0.05) and according to education level (*p* > 0.05). Not surprisingly, food safety training had little association with the dimensions, including food safety violations. Food handlers with training showed lower scores for exhaustion and cynicism and higher scores for dedication. Food handlers with leadership roles and managerial tasks presented higher scores on task crafting than other food handlers (4.69; 0.80 × 3.84; 1.0; *p* < 0.001). No differences were found between roles in job demands, job resources, burnout, and work engagement. Based on this finding and the fact that all subjects were classified as “food handlers”, the entire sample (n = 302) was included in the further steps. 

Table 4 shows the correlation coefficients. These results were mostly consistent with our theoretical expectations. Food safety violations were positively related to burnout and job demands and negatively related to job crafting, job resources, and work engagement. Weak to moderate correlations among the constructs could be observed, indicating a relationship between them and the absence of collinearity.

Table 5 shows that the factor loadings of the variables observed in their respective latent variables were larger than the factor loadings observed relative to the other latent variables. Thus, the discriminant validity of the model was adequate.

### 4.3. Structural Model

**Figure 2:**
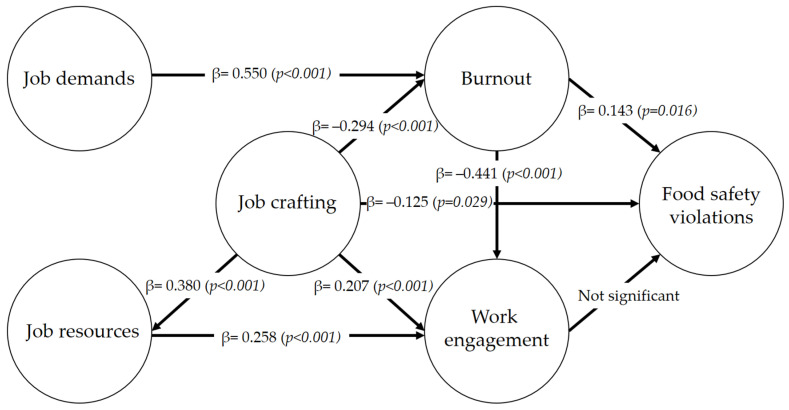
Final inner path model. The numbers represent the path coefficient values (β), and the numbers within parentheses represent the *p*-values of the t-statistics (based on bootstraps with 5000 samples).

After checking the reliability and validity of the measurement model, the structural model was evaluated. First, the path coefficients (β) were evaluated, and the results are shown in Figure 2. Hypotheses H1, H2, H3, H4a, H4b, H4c, H5, and H6 were confirmed by the structural model analysis. Hypothesis H7 was not confirmed. In this sense, burnout was strongly driven by job demands (H1: β = 0.550; *p* < 0.001; f^2^ = 0.53). Job resources was a positive predictor of work engagement (H2: β = 0.258; *p* < 0.001; f^2^ = 0.08). Burnout was a negative predictor of work engagement (H3: β = −0.411; *p* < 0.001; f^2^ = 0.22), thus confirming the base of the JD-R model. Job crafting was a positive predictor of job resources (H4a: β = 0.380; *p* < 0.001; f^2^ = 0.17) and work engagement (H4b: β = 0.207; *p* < 0.001; f^2^ = 0.07) and a negative predictor of burnout (H4c: β = −0.294; *p* < 0.001; f^2^ = 0.15). Food safety violations were predicted by job crafting (H5: β = −0.125; *p* = 0.029; f^2^ = 0.02) and burnout (H6: β = 0.143; *p* = 0.016; f^2^ = 0.02). 

Five percent of the variance of the construct food safety violations was explained by burnout and job crafting (R^2^ = 0.05). According to Cohen (1988), in the behavioral sciences, R^2^ = 0.02 is classified as a small effect, R^2^ = 0.13 as a medium effect and R^2^ = 0.26 as a large effect. A large explanatory power was found for burnout (R^2^ = 0.44) and work engagement (R^2^ = 0.51), a medium one for job resources (R^2^ = 0.14), and a small one for food safety violations (R^2^ = 0.05). The predictive relevance (Q^2^_predict_) of each indicator was adequate (Q^2^_predict_ > 0.0). Since the minority of the root mean squared error values of PLS-SEM were lower than the linear model, the model was valid [92].

## 5. Discussion

### 5.1. General Discussion

This study aimed to evaluate job crafting and burnout as predictors of safe behavior in food production. In addition, the application of the JD-R model with its great flexibility in the context of food services was investigated. We found that the JD-R model adapted well to the foodservice context, as all its hypotheses were confirmed (H1 to H4). According to Baker et al., burnout develops in a scenario where there is an imbalance between demands and resources at work [34]. On the other hand, work engagement is decreased by burnout [45,46,47,48] and increased by resources [42,43,44]. Our results are consistent with the extensive literature in the field and support the application of the JD-R model in the foodservice context.

When considering the extension of the JD-R model, we found that burnout was a positive predictor (H5) and job crafting (H6) was a negative predictor of food safety violations. Hypothesis 7, which suggested a negative relationship between work engagement and food safety violations, was not confirmed. The literature supports burnout as a predictor of inappropriate work practices. For workers to perform their tasks appropriately and thoughtfully, they need mental health [66]**.** However, when exhausted, they may not have the energy to perform their jobs safely [93,94]. In addition, one of the dimensions of burnout, depersonalization [36]**,** can lead to detachment from customers, colleagues, and the work itself as a whole [95]**,** leading the employees to stop performing some critical activities because they feel it is no longer worth investing energy in them. In this case, food safety can be easily overlooked because it is not easily perceived by customers [96] and is not a priority for foodservice workers [18]**.** For example, a Chinese study on 453 restaurant chefs found a significant association between burnout and improper hand hygiene [97]. 

Job crafting is a situated activity, i.e., tied to the work context, so different forms and levels of job crafting manifest themselves in different contexts [29,98]. In this sense, the task-crafting dimension had the lowest factor loading, followed by cognitive crafting and relational crafting. This result confirms the standardization of tasks expected in the foodservice industry [71]. In food-handling work, several standards and regulations are non-negotiable. Such standardization is manifested in different ways, for example, in Brazil, through compliance with regulations requiring following manuals and standardized procedures [99] and internationally through applying the points described in the Codex Alimentarius [99]**.** In addition, to maintain a high standard of preparation, it is strongly recommended that foodservice workers follow technical data sheets to ensure preparation nutritional, sensory, and visual qualities [100,101]. Although standardization is important for maintaining quality in foodservice, it discourages foodservice workers from using their creativity to improve foodservice tasks or to prepare dishes using techniques different from those standardized. For the food handler to be creative in food safety, they need consistent education in this area. Otherwise, job crafting may be used to facilitate and neglect safe practices.

Despite the low factor loading for task crafting, we found high factor loadings for cognitive and relational crafting. In a qualitative study conducted in a foodservice network in Finland, one of the most frequently mentioned forms of job crafting was relational crafting, noted as “getting to know colleagues in person”, “befriending peers”, “spending free time with peers”, and “encouraging peers” [102]. Although some researchers ignore the dimension of cognitive crafting in job-crafting rating scales [52]**,** others affirm its importance. They argue that changing the way workers view their tasks or work represents significant changes in the development of their function and involves cognitive reorientation [103,104].

Finally, job resources were positively related to work engagement but not to food safety violations. We believe that resource allocation can partly explain this result. When conflicting results related to job demands–resources appeared in the literature [105,106,107], researchers sought to understand why an increase in resources was not necessarily related to employee performance. This ambivalence raised the question of the resource-allocation perspective [108,109]. This perspective states that allocating resources to achieve performance-related goals may result in fewer resources available to employees to achieve other goals, such as their well-being. It is also argued that employees differ in terms of when, where, and how resources are allocated, affecting the impact of resources at work [110]. Thus, resources are not always allocated where they are thought to be, often leading to different results than expected. Another important factor is the extent to which burnout has negatively impacted work engagement. Because these factors are closely related, this finding may also have helped weaken the link between work engagement and food safety violations.

### 5.2. Theoretical Implications

This study has some theoretical implications. First, as mentioned above, the adequacy of the JD-R model was observed in the context of foodservice industry in Brazil, qualifying the variables pace and amount of work, physical effort, and role conflict as reliable indicators of the construct of job demands in this scenario. In addition, the variables “relationship with superior”, “justice”, and “relationship with colleagues” were classified as possible indicators of job resources in the restaurant industry. 

The second theoretical implication was that job crafting and food safety violations had low explanatory power. Although the path coefficient for job crafting and burnout was not high for food safety violations, we can consider the result relevant. Food safety behavior in the workplace is a multifactorial construct influenced by countless other factors, both psychosocial and organizational [111]**,** such as risk perception [112], optimistic bias [113,114], food safety knowledge [115]**,** standards [116], food safety culture [13], and others, which divide the influence on safe food into myriad parts. 

The third theoretical implication is that this was the first study with PLS-SEM that used a risk measure as an outcome variable. Other studies that examined food safety violations used the number of violations observed in the service [14,17]. The use of health risk was interesting because it weighted each violation based on the risk of an outbreak. One limitation is that there are few validated tools for this purpose in different languages based on local regulations.

### 5.3. Practical Implications

From a practical perspective, our results suggest that the JD-R model has proven to be a useful tool for use in the context of food services. Managers in the foodservice industry, then, must be constantly vigilant to ensure that employee demands are in balance with resources so that food production can be accomplished safely. We also emphasize the importance of harmonious relationships with both supervisors and co-workers and the need for equity in the foodservice industry to maintain employees’ commitment to their work.

The second practical implication was a significant relationship of job crafting with job resources, work engagement, and food safety violations. We believe it is possible to improve task crafting without violating food safety regulations, e.g., by allowing workers to suggest recipes, preparation methods, ingredients, and work organization. In this sense, we propose that managers consider food handlers not only as hands, as was the case in Taylorism, but also as brains capable of actively revising their work.

Finally, employees are traditionally trained only in food safety practices. Our study has shown that attention must also be paid to the mental health of foodservice workers. Mental health and a positive environment are critical in keeping these workers from the spiral of loss of resources, maintaining their health, and performing well in food handling.

### 5.4. Limitations and Future Research

The study has some limitations. First, it is a cross-sectional study, so it is impossible to establish a causal relationship. In addition, this study design limits how burnout, job crafting, and work engagement evolve throughout different routines. Second, we chose a study design focusing on organizational variables related to the JD-R model. Merging this model with variables known to affect food safety behaviors (e.g., food safety climate, culture, leadership) may help predict the true effect size and possible moderating effects of the former over the latter.

Another limitation is that many individual characteristics could affect the results. We develop a general model to predict food safety behavior. However, future studies could incorporate a multigroup hypothesis to compare different roles, education, and experience related to food safety.

We evaluate job crafting based on respondents’ perceptions. However, it would be interesting to evaluate the limits and possibilities of task crafting considering health legislation, which is non-negotiable. In this case, it is necessary to determine which practices foodservice workers suggest can be incorporated into the routine. It would also be interesting to verify the extent to which culinary skills and experience with different cooking types and utensils can favor good practices through motivational logic and meaning at work.

## 6. Conclusions

In light of the JD-R model, this study aimed to examine the constructs of job crafting, burnout, and work engagement as predictors of safe food-production behaviors measured as food safety violations. It was found that burnout could predict unsafe food-production behaviors, suggesting that food companies need to provide emotional support to their employees. On the other hand, job crafting, especially relational crafting, and cognitive crafting contributed to increased work engagement and safe behavior in food production, thus proving to be a promising construct for coping with FBDs. Results also demonstrated the applicability of the JD-R model to the food-production context. Thus, it was predicted that an increase in job resources led to better coping with job demands, increasing employee engagement. Increased job demands led to burnout, leading to a decrease in engagement. Finally, such results highlighted the need to give employees more autonomy to actively revise their work. These findings show the promise of psychosocial and organizational variables for a better understanding of safe food-handling behavior.

## Figures and Tables

**Table 1:** Mean violations and risk score of assessed restaurants.

Section	Mean Violations (%)	Mean Risk Score	Range of Risk Score
1—Water supply	3.2	3.18	0.0 to 34.7
2—Construction, facilities, equipment, furniture, and utensils	36.3	33.30	0.0 to 90.53
3—Sanitization of facilities, equipment, furniture, and utensils	55.4	143.18	0.0 to 278.0
4—Integrated control of disease vectors and urban pests	36.3	3.96	0.0 to 12.47
5—Food handlers	77.3	65.8	0.0 to 124.45
6—Raw materials, ingredients, and packaging	40.9	91.27	0.0 to 236.13
7—Food preparation	51.3	350.26	0.0 to 895.0
8—Storage and transport of prepared food	29.5	109.76	0.0 to 827.33
9—Documents *	50.0	*	*

* Items from this section do not have a risk score. Please see Da Cunha et al., 2014 [84], for more details about the risk score.

**Table 2:** Socioeconomic and labor characterization of foodservice workers.

Variable	Category	n	(%)
Sex	Men	155	51.32
	Women	147	48.68
Education level	Incomplete primary education	25	8.27
	Complete primary education	23	7.62
	Incomplete high school	72	23.84
	Complete high school	143	47.35
	Incomplete higher education	22	7.28
	Complete higher education	17	5.63
Income	Up to BRL 1,212.00	3	0.99
	BRL 1,212.01 to BRL 2,242.00	261	86.42
	BRL 2,424.01 to BRL 4,848.00	35	11.59
	BRL 4,848.01 to BRL 7,272.00	2	0.66
	More than BRL 7,272.00	1	0.33
Role	Junior chef	67	22.19
	Kitchen porter	121	40.07
	Dishwasher	41	13.58
	Stockist	9	2.98
	Head chef	6	1.99
	Waiter	27	8.94
	Manager	19	6.29
	Other	12	3.97
Had food safety training?	No	106	35.10
	Yes	196	64.90

**Table 3:** Results of analysis of construct means, reliability, convergent validity, and multicollinearity.

Construct/Dimensions	Mean (SD)	Factor Loading	VIF	AVE	CR
**Job crafting**	4.14 (0.72)	-	-	0.494	0.735
Task crafting	3.90 (1.07)	0.436	1.12		
Cognitive crafting	4.80 (0.91)	0.875	1.17		
Relational crafting	3.71 (1.02)	0.708	1.12		
**Job demands**	3.50 (0.96)	-	-	0.538	0.773
Role conflict	2.46 (0.81)	0.570	1.06		
Pace and amount work	4.99 (1.39)	0.748	1.33		
Physical effort	3.05 (1.60)	0.854	1.32		
**Job resources**	5.98 (0.97)	-	-	0.545	0.773
Relationship with superior	6.31 (1.15)	0.848	1.35		
Justice	5.80 (1.49)	0.777	1.31		
Relationship with colleagues	5.85 (1.37)	0.559	1.07		
**Burnout**	1.57 (0.87)	-	-	0.601	0.813
Professional efficacy	0.25 (0.28)	0.612	1.11		
Cynicism	1.42 (1.23)	0.857	1.71		
Exhaustion	3.00 (1.59)	0.833	1.61		
**Work engagement**	4.91 (0.92)	-	-	0.708	0.879
Vigor	4.96 (1.07)	0.892	2.32		
Dedication	4.80 (1.24)	0.894	2.35		
Absorption	4.96 (0.99)	0.728	1.32		
**Food safety violations**		-	-		
Food handlers	58.2 (1.0)	0.914	1.90	0.743	0.897
Food preparation	304.8 (215.1)	0.931	2.73		
Raw materials, ingredients, and packaging	80.3 (46.9)	0.777	1.95		

SD = standard deviation; VIF = variance inflation factor; AVE = average variance extracted; CR = composite reliability. Range: job crafting = 1 to 6; job demands and job resources = 1 to 7; burnout and work engagement = 0 to 6.

**Table 4:** Correlation among the constructs.

Constructs	1	2	3	4	5
1—Burnout	1.000				
2—Job crafting	−0.367	1.000			
3—Job demands	0.603	−0.131	1.000		
4—Job resources	−0.581	0.382	−0.355	1.000	
5—Work engagement	−0.627	0.478	−0.349	0.580	1.000
6—Food safety violations	0.185	−0.127	0.170	−0.156	−0.204

**Table 5:** Measurements of model loadings and cross-loadings.

Dimension	Burnout (B)	Job Crafting (JC)	Job Demands (JDs)	Job Resources (JRs)	Food Safety Violation (FSV)	Work Engagement (WE)
Cynicism (B1)	**0.857**	−0.296	0.491	−0.528	0.117	−0.528
Professional inefficacy (B2)	**0.612**	−0.345	0.250	−0.369	0.119	−0.484
Exhaustion (B3)	**0.833**	−0.246	0.598	−0.451	0.200	−0.481
Cognitive crafting (JC1)	−0.406	**0.875**	−0.108	0.332	−0.125	0.450
Task crafting (JC2)	−0.007	**0.436**	0.096	0.119	−0.035	0.116
Relational crafting (JC3)	−0.198	**0.708**	−0.182	0.285	−0.185	0.279
Role conflict (JD1)	0.343	−0.214	**0.570**	−0.270	0.095	−0.237
Pace and amount work (JD2)	0.361	−0.042	**0.748**	−0.239	0.106	−0.205
Physical effort (JD3)	0.557	−0.087	**0.854**	−0.281	0.157	−0.314
Relationship with superior (JR1)	−0.458	0.334	−0.280	**0.848**	−0.140	0.523
Justice (JR2)	−0.509	0.232	−0.290	**0.777**	−0.155	0.442
Relationship with colleagues (JR3)	−0.315	0.275	−0.217	**0.559**	−0.059	0.276
Raw materials, ingredients, and packaging (FSV1)	0.104	−0.153	0.075	−0.057	**0.777**	−0.129
Food handlers (FSV2)	0.228	−0.173	0.215	−0.204	**0.914**	−0.221
Food preparation (FSV3)	0.137	−0.139	0.120	−0.142	**0.931**	−0.180
Dedication (WE1)	−0.576	0.402	−0.316	0.482	−0.204	**0.892**
Absorption (WE2)	−0.420	0.348	−0.230	0.458	−0.139	**0.728**
Vigor (WE3)	−0.601	0.407	−0.330	0.518	−0.180	**0.894**

Bold values are the loadings with the highest values among the constructs.

## Data Availability

Data may be provided by the corresponding author upon request.
